# Left behind: widening disparities for males and females in US county life expectancy, 1985–2010

**DOI:** 10.1186/1478-7954-11-8

**Published:** 2013-07-10

**Authors:** Haidong Wang, Austin E Schumacher, Carly E Levitz, Ali H Mokdad, Christopher JL Murray

**Affiliations:** 1 Institute for Health Metrics and Evaluation, University of Washington, 2301 5th Avenue, Suite 600, Seattle, WA 98121, USA

## Abstract

**Background:**

The United States spends more than any other country on health care. The poor relative performance of the US compared to other high-income countries has attracted attention and raised questions about the performance of the US health system. An important dimension to poor national performance is the large disparities in life expectancy.

**Methods:**

We applied a mixed effects Poisson statistical model and Gaussian Process Regression to estimate age-specific mortality rates for US counties from 1985 to 2010. We generated uncertainty distributions for life expectancy at each age using standard simulation methods.

**Results:**

Female life expectancy in the United States increased from 78.0 years in 1985 to 80.9 years in 2010, while male life expectancy increased from 71.0 years in 1985 to 76.3 years in 2010. The gap between female and male life expectancy in the United States was 7.0 years in 1985, narrowing to 4.6 years in 2010. For males at the county level, the highest life expectancy steadily increased from 75.5 in 1985 to 81.7 in 2010, while the lowest life expectancy remained under 65. For females at the county level, the highest life expectancy increased from 81.1 to 85.0, and the lowest life expectancy remained around 73. For male life expectancy at the county level, there have been three phases in the evolution of inequality: a period of rising inequality from 1985 to 1993, a period of stable inequality from 1993 to 2002, and rising inequality from 2002 to 2010. For females, in contrast, inequality has steadily increased during the 25-year period. Compared to only 154 counties where male life expectancy remained stagnant or declined, 1,405 out of 3,143 counties (45%) have seen no significant change or a significant decline in female life expectancy from 1985 to 2010. In all time periods, the lowest county-level life expectancies are seen in the South, the Mississippi basin, West Virginia, Kentucky, and selected counties with large Native American populations.

**Conclusions:**

The reduction in the number of counties where female life expectancy at birth is declining in the most recent period is welcome news. However, the widening disparities between counties and the slow rate of increase compared to other countries should be viewed as a call for action. An increased focus on factors affecting health outcomes, morbidity, and mortality such as socioeconomic factors, difficulty of access to and poor quality of health care, and behavioral, environmental, and metabolic risk factors is urgently required.

## Background

United States life expectancy at birth ranks 40th for males and 39th for females across 187 countries in the world in 2010 [1]. Given that the US spends more than any other country on health care [2-5] the poor relative performance of the US compared to other high-income countries has attracted increasing attention [6,7]. An important dimension to poor national performance is the large disparities in life expectancy and other metrics of mortality across populations within the US [8-12]. Racial and ethnic disparities as well as socio-economic disparities are large [13-15]. Multiple studies have demonstrated large variation in life expectancy across US counties [9]. Understanding large disparities in life expectancy within the US is important in its own right but may also provide insights into poor national performance.

Past evidence has investigated disparities in life expectancy at birth in the US. Ezzati et al. [8] reported not only that there were large disparities in life expectancy across counties in the US but from 1983 to 1999, female life expectancy fell in 180 counties and male life expectancy fell in 11 counties. Kulkarni et al. [11] reported that from 2000 to 2007, many US counties fell increasingly behind the levels achieved in high-income countries with the best outcomes. Kindig and Cheng [16] found evidence that mortality increases occurred from 1992–1996 to 2002–2006 for females in 42% of US counties. These reversals for females in life expectancy are cause for broad concern especially coming on top of large pre-existing disparities in the US. Speculation on the causes of these reversals include the impact of tobacco consumption in females, rising levels of obesity, and associations in rates of change with a range of socio-economic factors [16]. Tracking the evolution of US disparities following the 2008 financial crisis is important, especially for females.

In this paper, we examine trends in life expectancy at the county level from 1985 to 2010. We take advantage of the release of the 2010 Census age structure by county and updates for the intercensal period 2000 to 2010. Further, demographic estimation methods that more accurately reflect uncertainty have been recently widely applied [1] and have been incorporated into this study. Combined with new county mortality data through 2010, we are able to examine long-term and recent trends in county life expectancy for males and females.

## Methods

We applied a statistical model to estimate age-specific mortality and life expectancy by age for US counties for the years 1985 to 2010, the last year with available mortality data at the county level. Our methodology requires five years of mortality data prior to each year estimated to make robust estimates. In addition, we need a set of counties or county aggregates that we can map and match to prior years in order to estimate a coherent time trend. These requirements mean that we are only able to estimate a county time series from 1985 to 2010.

### Modeling approach

Estimating health outcomes for small areas is challenging as researchers are faced with large stochastic fluctuations due to small numbers of events or small numbers sampled. Commonly used methods to deal with these issues include pooling multiple years of data, borrowing strength across geospatial units, or using structured relationships with covariates [17]. Kulkarni et al. proposed a method for county life table estimation that integrates these three approaches [11], which we use here. Briefly, we used a mixed effects Poisson regression with time, geospatial, and covariate components.

The model is specified below:

$$\begin{array}{l} \ln y_{\mathrm{rjt}}=\ln P_{\mathrm{rjt}}+\beta_{0}+\beta_{1}\cdot {\text{income}}_{\mathrm{jt}}+\beta_{2}\cdot {\mathrm{education}}_{\mathrm{jt}}\\ \;+\beta_{3}\cdot \sigma_{{\mathrm{post}}_{j}}+\beta_{4}\cdot {\mathrm{race}}_{\mathrm{jt}}+\left(\beta_{5}+\gamma_{j}\right)\cdot {\mathrm{time}}_{t}+\mu_{j}\\ \;+\epsilon_{\mathrm{rjt}} \end{array}$$

where *yrjt* and *P*_*ijt*_ are the death count and population for race *r* within county *j* in year *t*. *Incomejt* is county per capita income for year *t*. *Educationjt* is the percent of adults within county *j* having completed high school in each year. *Race*_*jt*_ is a categorical variable for three race groups (white, black, and other). Asians and Native Americans were grouped into a single category to reduce the sensitivity of the model to known racial miscoding in population and death counts. *σpostj* is the geospatial component, calculated as the average of the posterior model county random intercept for counties adjacent to county *j* to account for residual spatial patterns. The values for *σpostj* are derived from running as a prior step the same model without the geospatial component to derive the posterior values of the county random effect. *μj* is the posterior value of the county random intercept. *Time*_*t*_ is the calendar year of mortality, and γj is a random slope on time for each county. This specification allows mortality in each county to have a unique trend. The county population size affects the contribution of the random components on death counts, leading to more emphasis on recorded death counts when predicting mortality for larger counties. The model was estimated separately by sex and five-year age groups because the magnitude of the county random effect varies by age. Because larger counties have observed age-specific death rates with narrower uncertainty intervals than derived from the model, we use the output of the mixed effects logistic regression of counties with non-zero death counts in all years for an age-sex group over the entire 1985–2010 period as a prior mean function for a Gaussian Process Regression where hyper-parameters were adapted from the those used by Wang et al. [1] for high-income countries. The effect of this step is to more accurately reflect the uncertainty in age-specific death rates in large counties.

The outcome of the analysis is a predicted age-, sex-, and race-specific death count for each county in the year of analysis. We used these counts, together with corresponding population figures, to calculate sex-specific life expectancy for each county. We used the method proposed by Wang et al. [1] to estimate the years lived in the terminal age group of the life table. With an increasing proportion of the population surviving to older age groups, accurately estimating age-specific mortality rate in people 85 or older is becoming crucial for estimating life expectancy at birth accurately. When we examine both the mean and uncertainty interval of the relative error in predicted age-specific mortality rates, the method developed by Wang et al. has been shown to provide results with much less bias when comparing to other widely used extrapolation methods [1].

To produce estimates for a given calendar year, we used data for that year and the five years prior to estimate the mixed effects Poisson regression. Uncertainty in county life expectancy was calculated using simulations by drawing repeatedly from the posterior distributions of the sex-, race-, age-, and county-specific death counts if the age-sex group in a county did not meet the criteria for Gaussian Process Regression and by Markov Chain Monte Carlo methods if it did meet the criteria.

We used mortality data, including county of residence, sex, race, age, and year of death from the National Center for Health Statistics (NCHS). County population denominators broken down by age, race, sex, and year were taken from the National Census Bureau for years prior to 1990 and from NCHS bridged-race population estimates otherwise. Our estimates of per-capita income were taken from the US Bureau of Economic Analysis. The series was deflated to generate real income per capita using GDP deflators provided by the World Bank. Educational attainment was based on census data from 1980, 1990, and 2000 and American Community Surveys for 2009–2011. Values for intervening years were based on linear interpolation.

The 3,143 US county equivalents were arranged into 2,356 merged county clusters, each consisting of a single county or multiple counties. The merging was done to account for changes in county boundaries over time to ensure consistency and to overcome the fact that some counties had very small numbers of deaths. National results were calculated directly from the raw data.

## Results

For the US as a nation, female life expectancy increased from 78.0 years in 1985 to 80.9 years in 2010, and male life expectancy increased from 71.0 years in 1985 to 76.3 years in 2010. In 1985, the gap between female and male life expectancy was 7.0 years; this has narrowed progressively beginning in 2002 to only 4.6 years in 2010. The slower rates of improvement in female life expectancy, and consequently the narrowing gap, is consistent with the worsening national rank for female life expectancy across 187 countries (from 19 in 1985 to 39 in 2010), while the rank for males over this period changed from 29 to 40 [1].

**Figure 1:**
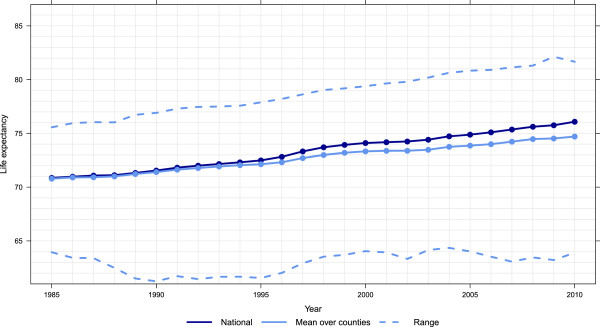
National, mean of county, and range of life expectancy, males, 1985–2010.

**Figure 2:**
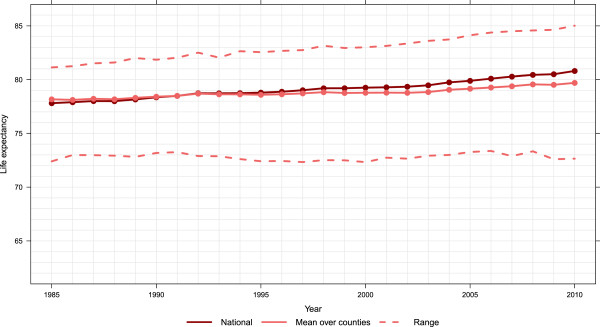
National, mean of county, and range of life expectancy, females 1985–2010.

Figure 1 shows trends in US life expectancy as well as the mean life expectancy across counties and the highest and lowest life expectancy in each year. For males in Figure 1, the highest life expectancy has steadily increased from 75.5 in 1985 to 81.7 in 2010, 0.25 years per calendar year. The lowest life expectancy remains below 65 throughout the entire 25-year period. For females, as seen in Figure 2, the highest life expectancy has increased 0.16 years per calendar year, from 81.1 to 85.0. The lowest life expectancy for females has remained relatively constant around 73 over the entire 25-year period. By 2010, the highest county-specific male life expectancy was greater than the female national life expectancy. The increasing difference in both Figures 1 and 2 between national life expectancy and the arithmetic mean of county-level life expectancy estimates indicates higher heterogeneity in life expectancy across counties. Moreover, it shows that an increasing number of counties have life expectancy at birth that are below the national values.

**Figure 3:**
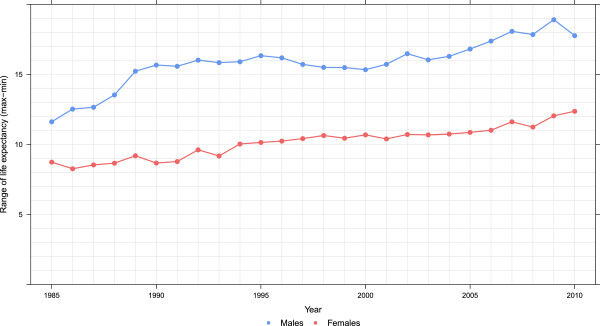
Range in life expectancy, males and females, 1985–2010.

**Figure 4:**
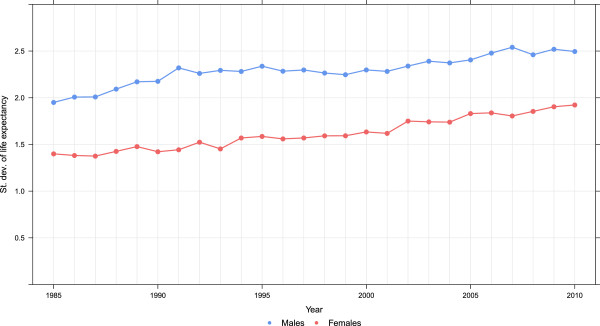
Standard deviation of life expectancy, males and females, 1985–2010.

The expansion of disparities in county life expectancy throughout the period visible in Figures 1 and 2 is quantified using two metrics in Figures 3 and 4: the difference between the maximum and minimum life expectancy and the standard deviation of life expectancy across counties. These are computed separately for males and females. For males, there appears to have been three phases evident in both metrics of inequality: a period of rising inequality from 1985 to 1993, a period of stable inequality from 1993 to 2002, and rising inequality from 2002 to 2010. For females, in contrast, inequality has steadily increased during the 25-year period. Of note, both metrics show that there is greater inequality in male life expectancy across counties than for females. As of 2010, female life expectancy at birth in Marin County, CA is 85.0 years (95% uncertainty interval: 84.5, 85.6). In Perry County, KY, it is 72.7 years (71.3, 73.8), a gap of 12.3 years. For males, Fairfax County, VA had the highest life expectancy of 81.7 years (81.3, 82.0), while in McDowell County, WV it was 63.9 years (62.0,65.6), a gap of 17.8 years. Even within a state there are wide disparities. For example, females in Loudon County, VA have the 12th-highest life expectancy at 84.2 years (83.5, 84.8), while in Petersburg County, VA females have the fifth-lowest at 73.7 years (72.1, 75.2).

**Figure 5:**
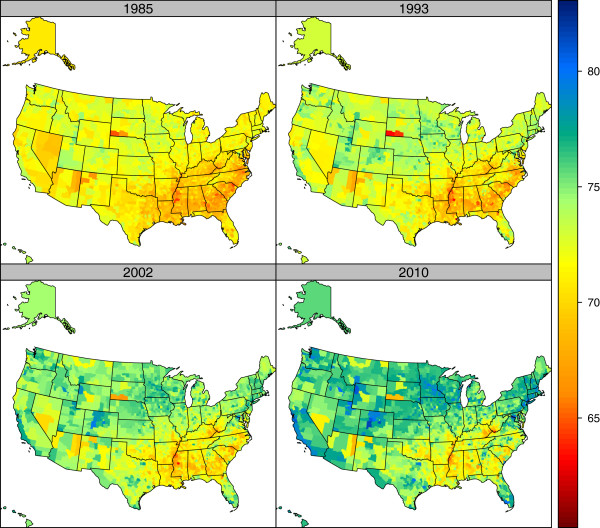
Life expectancy by county, males, 1985, 1993, 2002, and 2010.

**Figure 6:**
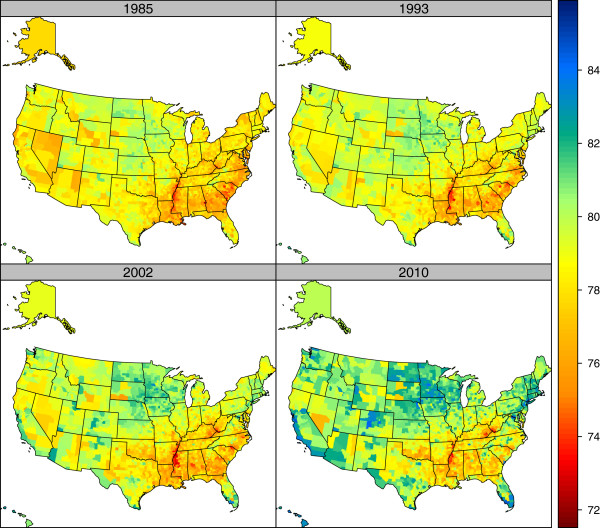
Life expectancy by county, females, 1985, 1993, 2002, and 2010.

Figures 5 and 6 show maps of US county life expectancy separately for males and females at four points: 1985, 1993, 2002, and 2010. These years correspond to apparent changes in the trends in disparities across US counties, especially for males. Some general patterns are evident. In all time periods, the lowest life expectancies are seen in the South, the Mississippi basin, West Virginia, Kentucky, and selected counties in the West and Midwest that have large Native American reservation populations. However, the maps reveal dramatic changes that have occurred unevenly across the US. Substantial improvements in life expectancy are seen in multiple locations: parts of California, most of Nevada, Colorado, rural Minnesota, Iowa, and parts of the Dakotas, some Northeastern states, and parts of Florida. The improvements tend to occur in the same locations for males and for females. Table 1 lists the counties in the US with the highest and lowest life expectancies in 2010. Table 2 lists the counties with the largest increases and decreases between 1985 and 2010. The largest increases in life expectancy over the 25-year period for females were in four New York City counties, Marin and San Francisco counties in California, and in counties in Colorado, Wyoming, South Carolina, and New Jersey. For males, a number of counties in or near New York City along with multiple counties in Virginia saw the largest gains. Ten of the worst-performing counties for females (with declines in life expectancy) were in Oklahoma, and five were in Kentucky. For males, the worst performers were in Kentucky, Oklahoma, Mississippi, and Alabama.

**Table 1:** Top 20 and bottom 20 counties in terms of life expectancy by sex, 2010

**Top counties**						**Bottom counties**			
**Rank (top)**	**Name**	**Life expectancy**	**Lower**	**Upper**	**Rank (bottom)**	**Name**	**Life expectancy**	**Lower**	**Upper**
Females									
1	Marin, California	85.02	84.46	85.56	1	Perry, Kentucky	72.65	71.31	73.79
2	Montgomery, Maryland	84.87	84.53	85.19	2	McDowell, West Virginia	72.90	71.37	74.29
3	Collier, Florida	84.62	84.09	85.10	3	Tunica, Mississippi	73.36	71.69	74.63
4	Santa Clara, California	84.54	84.29	84.80	4	Quitman, Mississippi	73.36	71.69	74.63
5	Fairfax County, Virginia	84.52	84.19	84.84	5	Petersburg, Virginia	73.69	72.11	75.19
6	San Francisco, California	84.38	84.02	84.73	6	Sunflower, Mississippi	73.85	72.26	75.16
7	Gunnison, Colorado	84.33	83.04	85.47	7	Mississippi, Arkansas	73.85	72.70	74.95
8	Pitkin, Colorado	84.33	83.04	85.47	8	Mingo, West Virginia	73.92	72.79	74.95
9	San Mateo, California	84.30	83.94	84.70	9	Washington, Mississippi	74.09	72.93	75.19
10	Bergen, New Jersey	84.26	83.95	84.56	10	Leslie, Kentucky	74.12	72.96	75.16
11	Douglas, Colorado	84.17	83.56	84.77	11	Clay, Kentucky	74.12	72.96	75.16
12	Loudoun, Virginia	84.16	83.54	84.77	12	Bolivar, Mississippi	74.32	73.08	75.45
14	Stearns, Minnesota	84.13	83.42	84.82	13	Phillips, Arkansas	74.44	72.91	75.71
13	Lincoln, South Dakota	84.11	82.94	85.24	14	Logan, West Virginia	74.50	73.35	75.66
15	New York, New York	84.09	83.83	84.35	15	Coahoma, Mississippi	74.56	73.09	75.80
16	Westchester, New York	84.05	83.75	84.36	16	Holmes, Mississippi	74.59	73.06	75.93
17	Brown, Minnesota	83.86	82.66	84.87	17	Wyoming, West Virginia	74.79	73.47	75.97
18	Los Alamos, New Mexico	83.86	82.62	85.05	18	Harlan, Kentucky	74.86	73.62	75.89
19	Orange, California	83.82	83.62	84.02	19	Haralson, Georgia	74.89	73.69	75.99
20	Cedar, Nebraska	83.81	82.48	85.01	20	Franklin, Alabama	74.92	73.80	75.91
Males									
1	Fairfax County, Virginia	81.67	81.32	82.02	1	McDowell, West Virginia	63.90	62.04	65.61
2	Gunnison, Colorado	81.65	80.39	82.84	2	Bolivar, Mississippi	65.03	63.52	66.46
3	Pitkin, Colorado	81.65	80.39	82.84	3	Perry, Kentucky	66.52	65.15	67.73
4	Montgomery, Maryland	81.57	81.23	81.91	4	Floyd, Kentucky	66.59	65.22	67.86
5	Marin, California	81.44	80.91	82.01	5	Tunica, Mississippi	66.70	65.18	68.04
6	Douglas, Colorado	81.41	80.77	82.01	6	Quitman, Mississippi	66.70	65.18	68.04
7	Eagle, Colorado	81.01	79.83	82.18	7	Sunflower, Mississippi	66.92	65.57	68.33
8	Loudoun, Virginia	81.00	80.37	81.65	8	Coahoma, Mississippi	66.92	65.32	68.49
9	Santa Clara, California	80.98	80.69	81.25	9	Washington, Mississippi	67.10	65.75	68.50
10	Teton, Wyoming	80.93	79.85	81.84	10	Macon, Alabama	67.19	65.71	68.55
11	Los Alamos, New Mexico	80.82	79.51	81.95	11	Bullock, Alabama	67.19	65.71	68.55
12	Bergen, New Jersey	80.53	80.22	80.86	12	Mingo, West Virginia	67.26	65.91	68.57
13	Howard, Maryland	80.41	79.79	80.98	13	Phillips, Arkansas	67.36	65.78	68.81
14	Leelanau, Michigan	80.41	79.22	81.41	14	Wyoming, West Virginia	67.47	66.03	68.76
15	Arlington, Virginia	80.39	79.76	81.11	15	Owsley, Kentucky	67.50	66.01	68.86
16	Falls Church, Virginia	80.39	79.76	81.11	16	Breathitt, Kentucky	67.50	66.01	68.86
17	San Mateo, California	80.34	79.98	80.71	17	Pike, Kentucky	67.50	66.36	68.57
18	Somerset, New Jersey	80.18	79.70	80.66	18	Petersburg, Virginia	67.79	66.24	69.17
19	Summit, Colorado	80.09	79.19	80.82	19	Holmes, Mississippi	67.87	66.19	69.45
20	Collier, Florida	80.08	79.51	80.65	20	Sharkey, Mississippi	67.95	65.85	69.67

**Table 2:** Top 20 and bottom 20 counties in terms of change in life expectancy by sex, 1985-2010

**Top counties**					**Bottom counties**				
**Rank (top)**	**Name**	**Change in life expectancy**	**Lower**	**Upper**	**Rank (bottom)**	**Name**	**Change in life expectancy**	**Lower**	**Upper**
Females									
1	New York, New York	8.37	7.91	8.79	1	Fayette, Alabama	−3.47	−5.41	−1.71
2	Loudoun, Virginia	7.77	6.59	8.99	2	Harmon, Oklahoma	−3.39	−5.07	−1.6
3	Kings, New York	6.7	6.37	7.03	3	Beckham, Oklahoma	−3.39	−5.07	−1.6
4	Bronx, New York	6.39	5.91	6.85	4	Leslie, Kentucky	−3.17	−4.75	−1.59
5	Gunnison, Colorado	6.28	4.58	7.91	5	Clay, Kentucky	−3.17	−4.75	−1.59
6	Pitkin, Colorado	6.28	4.58	7.91	6	Seminole, Oklahoma	−2.73	−4.35	−1.13
7	Marin, California	6.27	5.47	7.07	7	Haralson, Georgia	−2.58	−4.46	−0.89
8	Prince William, Virginia	6.09	5.02	7.13	8	Murray, Oklahoma	−2.58	−4.06	−1.17
9	San Francisco, California	6.05	5.52	6.61	9	Garvin, Oklahoma	−2.58	−4.06	−1.17
10	Beaufort, South Carolina	6.02	4.78	7.28	10	Perry, Kentucky	−2.57	−4.34	−0.92
11	Queens, New York	6.01	5.69	6.35	11	Johnston, Oklahoma	−2.52	−4.38	−0.78
12	St. Johns, Florida	5.94	4.87	7.14	12	Coal, Oklahoma	−2.52	−4.38	−0.78
14	Teton, Wyoming	5.8	3.82	7.72	13	Pontotoc, Oklahoma	−2.5	−4.26	−0.8
13	Douglas, Colorado	5.75	4.3	7.29	14	Tillman, Oklahoma	−2.43	−3.98	−0.82
15	Hudson, New Jersey	5.73	5.11	6.29	15	Jefferson, Oklahoma	−2.43	−3.98	−0.82
16	Rockland, New York	5.72	5.01	6.52	16	Cotton, Oklahoma	−2.43	−3.98	−0.82
17	Alexandria, Virginia	5.59	4.4	6.84	17	Walker, Alabama	−2.34	−3.81	−1.03
18	Nassau, New York	5.51	5.11	5.89	18	Whitley, Kentucky	−2.3	−3.89	−0.72
19	Pike, Pennsylvania	5.5	5.07	5.93	19	Casey, Kentucky	−2.29	−4.11	−0.72
20	Alameda, California	5.5	3.88	6.98	20	Marion, Alabama	−2.27	−3.75	−0.73
Males									
1	New York, New York	12.97	12.55	13.41	1	Floyd, Kentucky	−1.49	−3.23	0.3
2	San Francisco, California	10.6	10.05	11.18	2	McDowell, West Virginia	−1.45	−3.62	0.75
3	Kings, New York	9.76	9.39	10.12	3	Bolivar, Mississippi	−0.98	−2.91	1.1
4	Loudoun, Virginia	9.59	8.51	10.75	4	Perry, Alabama	−0.87	−2.76	1.27
5	Bronx, New York	9.57	9.08	10.1	5	Hale, Alabama	−0.87	−2.76	1.27
6	District of Columbia	9.37	8.67	10.09	6	Creek, Oklahoma	−0.69	−2.1	0.74
7	Forsyth, Georgia	9.16	7.71	10.74	7	Wyoming, West Virginia	−0.65	−2.44	1.27
8	Goochland, Virginia	9.15	7.51	10.89	8	Cherokee, Kansas	−0.56	−2.3	1.19
9	Alexandria, Virginia	8.84	7.48	10.13	9	Grundy, Tennessee	−0.55	−2.88	1.5
10	Hudson, New Jersey	8.63	8.06	9.23	10	Danville, Virginia	−0.36	−1.99	1.34
11	Queens, New York	8.5	8.12	8.88	11	Aransas, Texas	−0.34	−2.15	1.44
12	Colusa, California	8.45	6.54	10.5	12	Pike, Kentucky	−0.31	−1.81	1.09
13	Suffolk, Virginia	8.34	6.86	9.87	13	Owsley, Kentucky	−0.23	−2.09	1.79
14	Collier, Florida	8.19	7.19	9.26	14	Breathitt, Kentucky	−0.23	−2.09	1.79
15	Sumter, Florida	8.13	6.73	9.69	15	Benton, Tennessee	−0.19	−2.3	2.04
16	Rockwall, Texas	8.08	6.56	9.77	16	Mingo, West Virginia	−0.09	−1.88	1.67
17	Gunnison, Colorado	8.02	6.22	9.92	17	Wolfe, Kentucky	0.01	−1.87	2.02
18	Pitkin, Colorado	8.02	6.22	9.92	18	Lee, Kentucky	0.01	−1.87	2.02
19	Alameda, California	8.01	7.56	8.45	19	Pawnee, Oklahoma	0.04	−1.8	1.94
20	Dawson, Georgia	8	6.18	9.83	20	Coahoma, Mississippi	0.06	−2.01	2.15

Over the three intervals, we have examined how many counties have observed statistically significant improvements in life expectancy, significant declines in life expectancy, or changes that were not statistically significant using a one-tailed test. Tables 3, 4 and 5 show the cross-tabulation of significant changes in male and female life expectancy for the three intervals. During the first interval, 42 counties saw declines for females and 32 for males. But nearly twice as many counties saw significant increases for males as for females; the vast majority of counties had no significant change for females. For the period from 1993 to 2002, male life expectancy declined significantly in only six counties, while it did so significantly in 300 for females. This marked difference is evident at the other end of the spectrum, where the number of counties with significant increases for males was 3.8 times higher than for females. This period of relatively good life expectancy outcomes for males in many counties has been followed by a period from 2002 to 2010 where outcomes for males and females have been more similar: 37 significant declines for females and nine for males, with 1,427 significant increases for females and 1,895 for males.

**Table 3:** Number of counties with significant changes in males vs. females, 1985-1993

	**Males**			
**Females**	**Significant increase**	**No significant change**	**Significant decrease**	**Total**
Significant Increase	632	147	7	786
No Significant Change	880	1411	24	2315
Significant Decrease	3	38	1	42
Total	1515	1596	32	3143

**Table 4:** Number of counties with significant changes in males vs. females, 1993-2002

	**Males**			
**Females**	**Significant Increase**	**No Significant Change**	**Significant Decrease**	**Total**
Significant Increase	573	33	0	606
No Significant Change	1612	624	1	2237
Significant Decrease	143	152	5	300
Total	2328	809	6	3143

**Table 5:** Number of counties with significant changes males vs. females, 2002-2010

	**Males**			
**Females**	**Significant increase**	**No significant change**	**Significant decrease**	**Total**
Significant Increase	1095	332	0	1427
No Significant Change	788	884	7	1679
Significant Decrease	12	23	2	37
Total	1895	1239	9	3143

**Figure 7:**
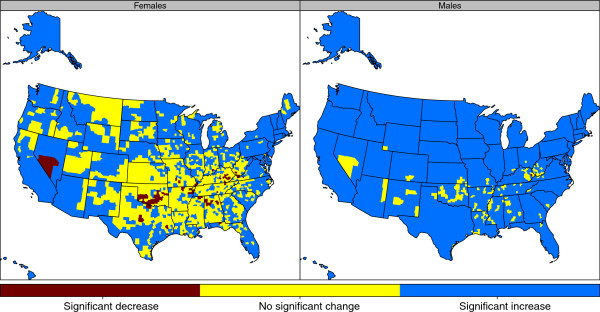
Significant changes in life expectancy by county, 1985–2010.

When we examine the changes in life expectancy at birth for the entire study period of 1985 to 2010, as shown in Figure 7, we observe significant decreases in life expectancy in only one county (Floyd County, KY) for males and 72 for females. The differences between males and females are more pronounced when we look at the counties with no significant change. For males, only 153 out of 3,143 counties have seen no significant change in life expectancy at birth. On the other hand, this number for females is 1,333 counties, or 42.4% of all counties in the US. Around 95.1% of the counties in the US have improved male life expectancy at birth from 1985 to 2010, while only just over half of all counties (55.3%) have seen improved female life expectancy at birth during the same time period.

**Figure 8:**
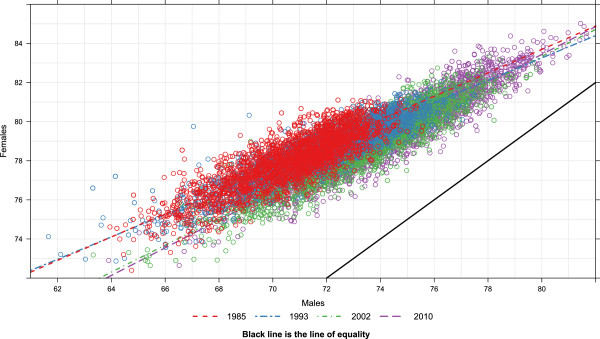
Life expectancy in females vs. males, 1985, 1993, 2002, and 2010.

Figure 8 compares male and female life expectancy by county at the same four points in time as before: 1985, 1993, 2002, and 2010. The figure demonstrates the high correlation between female and male life expectancy by county at each point in time. It also shows how the gap between male and female life expectancy is as wide as 11 to 13 years for the counties with the lowest life expectancies and as narrow as two to four years for counties with the highest life expectancies. From 1993 to 2002, the slope of the relationship between female and male life expectancy became steeper as many more female counties had declines in life expectancy compared to males. The steadily rising life expectancy for males and females in the best-performing counties over time is also evident, as is the lack of progress at the other end of the distribution.

**Figure 9:**
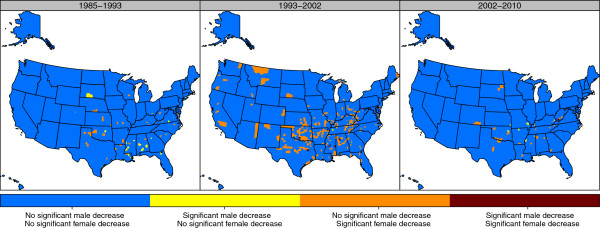
Significant decreases in life expectancy, stratified by sex, 1985–1993, 1993–2002, and 2002–2010.

Figure 9 shows the locations of counties that have had significant declines for males, females, or both in the three time periods. During the period 1993–2002, the large number of counties with female declines but not male declines is evident in a belt from Texas to West Virginia. In addition, significant declines for females were seen in a few counties in Western states, some with Native American reservations. During the most recent period, the limited number of counties with declines in life expectancy was still in the same zone from Texas to West Virginia.

We have also included an additional file with male and female life expectancy in every county across all years of our study (Additional file 1).

## Discussion

From 1985 to 2010, the US as a nation has seen improvements in life expectancy of 5.2 (5.2, 5.3) years for males and 3.0 (2.9, 3.0) years for females. However, these improvements are much less than what countries of similar income per capita have seen. Indeed, this slow pace of improvement has meant the global ranking of life expectancy in the US has fallen to 39 and 40 out of 187 nations for males and females, respectively. Faster growth in life expectancy for males than for females has narrowed the gender gap to 4.6 years in 2010. Examining these trends by county shows these national trends have occurred because of continued progress in expanding male and female life expectancy in a number of high-performing counties. The best counties have achieved female life expectancy of 85.0 and male life expectancy of 81.7 in 2010. At the same time, many counties have made no progress, or for the period 1993 to 2002, there have been declines for females in several hundred counties. Declines in life expectancy in some counties and stagnation in others means that inequality in life expectancy at birth among US counties increased dramatically from 1985 to 2010. In the last eight years, inequality in males has increased at an accelerated rate and remains consistently higher than for females despite the legacy of declining life expectancy from 1993 to 2002 for females in a substantial number of counties.

The reduction in the number of counties where female life expectancy is declining in the most recent period is welcome news. The decline in female life expectancy at birth in many counties from 1993 to 2002 needs explanation. Delayed peaks in female tobacco consumption compared to males may have been an important factor; for this period of time the impact of tobacco for males was declining and the impact for females was increasing. Lung cancer rates for females appear to have peaked around 2002 to 2005 at the national level [18-20]. Obesity is a major risk factor, with higher rates among females than males in many communities. Obesity levels increased in all counties but nine during the same time period [21]. Other studies have reported that the rates of increase in obesity at the national level may have also been slowing in the most recent time period [22,23]. Declines in female life expectancy were concentrated in the central part of the US, particularly in a belt from Texas to West Virginia; more detailed analyses would be helpful in understanding the concentration in these areas.

During the period covered by this study, life expectancy at birth among Organization for Economic Co-operation and Development (OECD) countries increased 4.45 years for males and 5.75 years for females [1]. Yet our study shows large segments of the US had no significant increases in life expectancy for females. In contrast, some counties in the US saw increases far above the OECD average. Understanding why there has been no progress for females in so many counties is an important priority requiring further research.

In addition, the stagnation in life expectancy in a substantial number of counties alongside improvements in life expectancy in many others has led to steadily widening inequalities. Despite the period of falling life expectancy for females in many counties, inequalities for males are larger than for females. The gap in life expectancy at birth between males and females steadily narrows as pace of improvement in male life expectancy has been much faster than that of females. This suggests that if the trends continue, differences between male and female life expectancy will eventually be much smaller than we currently observe. Indeed, these findings raise some serious questions to our public health systems. What are the causes for the steady increase in inequalities? Why are parts of America being left behind while some others are enjoying increasing gains in life expectancy?

To present our findings in perspective to changes occurring on the global level, we compared the life expectancy in the US counties in 2010 to those of other countries as calculated in the Global Burden of Diseases, Injuries, and Risk Factors Study 2010 [1]. The top counties for females, such as Marin County, CA and Montgomery County, MD, have life expectancies that rival some countries where people live the longest, such as Switzerland, Spain, and France. Montgomery, MD has a female life expectancy at birth that is slightly higher than Japan. For males, the top counties, such as Fairfax County, VA and Gunnison County, CO, have life expectancies higher than the top countries, such as Switzerland and Japan. Life expectancy for males in 11% and for females in 14% of US counties was below that of Nicaragua. In some counties, such as McDowell County, WV and Sunflower County, MS, life expectancies are lower than Bangladesh for males and Algeria for females. The complete failure by some communities to increase life expectancy from levels seen now in very poor countries likely has many distal and proximate causes. But most importantly, this slow progress should be viewed as a call for action to improve health and reduce inequalities in the US.

Stagnant or even declining life expectancies in some communities could be related to five types of factors: 1) migration; 2) socioeconomic factors such as poverty and education; 3) lack of access to health care; 4) poor quality of health care for those with access; and 5) behavioral, environmental, and metabolic risk factors. All could be operating simultaneously to explain some of the patterns observed at the county level. One explanation for the widening disparities could be that healthier individuals are migrating from disadvantaged communities, which could lower life expectancy in the community they left and raise it in the community they move to. Ezzati et al. [8] used Internal Revenue Service tax records that record movements from county to county to explore how much migration might explain disparities. They found that in general individuals moved from high life expectancy to low life expectancy communities and not the reverse. While their finding suggests migration may not be a major factor in the national patterns, it could be an important factor in selected counties that have experienced substantial in- or out-migration. On the other hand, net immigration of young Hispanic adults with lower mortality could have tended to increase life expectancy at birth for some counties and the nation as a whole.

Many studies have showed that socioeconomic factors are associated with poor health outcomes [24,25]. The patterns we report, however, cannot be simply explained by county-level changes in income per capita. During the period 1993 to 2002, US economic growth was associated with worsening inequality especially for women. Simple comparisons of change in life expectancy and change in income per capita at the county level for the period 1985 to 2010 show effectively no relationship. Mother’s education has a strong protective effect on child mortality [26-30]. Females with higher education are more likely to know the danger signs for their health and that of their families. They may be more likely to seek medical care and adhere to it. The Institute of Medicine report on shorter lives [31] has emphasized the importance of social factors including poverty and inequality rates in understanding poor overall outcomes in the US. Simple comparisons of change in county life expectancy and change in educational attainment in this analysis do not show the expected relationship. While income, education, and economic inequality are likely important factors, they are not the only determinants of outcomes; the consistently high life expectancies seen in rural and below-median-income counties in Minnesota, Iowa, and parts of the Dakotas indicate that there are more complex factors that may also be important.

In the US, many individuals lack health insurance or are underinsured, and lack of health insurance has been shown at the individual level to be associated with increased risk of poor health outcomes [32]. At the community level, however, it has been more difficult to show a relationship between insurance coverage and mortality outcomes [33]. Simple correlation analysis at the county level, however, does not show a relationship to life expectancy. The disconnect between the clear findings on insurance and individual outcomes and the lack of this association at the community level suggests other factors intervene that are more powerful determinants of poor outcomes at the community level. The quality of medical care is another determinant of health outcome [34]. However, this does not involve only medical errors. Good medical care ensures that patients are properly followed to receive treatment and that conditions are controlled. Appropriate management of key conditions such as elevated blood pressure varies substantially across counties [35].

Modifiable behavioral, environmental, and metabolic risk factors are critical determinants of health in the US and likely critical determinants of health at the community level. Levels of obesity, for example, are highly correlated with mortality and life expectancy [21,36]. Previous studies reported on the association between preventable risk factors and premature mortality [36,37]. Poor diet, physical inactivity, and smoking accounted for 51.8% of premature deaths in the US in 2010 [9,36]. Therefore, the biggest efforts should be to reduce these risk factors across the US. Unfortunately, data on the prevalence and changes of these risk factors at the county level are lacking.

Given the diversity of demography, epidemiology, physical infrastructure, and health system organization at the local level, a single national solution may not be the most effective for all risk factors. Different approaches should be implemented and evaluated. Indeed, prevention should be viewed as an investment, like retirement funds, where diversifying the portfolio is a key for success. A county health department or the federal government could then shift resources to interventions that are successful and stop funding what is not working.

Several studies have reported that medical practitioners have a key role in prevention [38,39]. Patients who received medical advice to reduce weight or stop smoking were more likely to attempt and achieve these behavior changes. Therefore, involving the medical system in prevention across the US is crucial. Hospitals and medical centers should be encouraged to be involved in prevention efforts in their community. The possible benefits (and costs) of introducing a system for grading and reimbursing health facilities based in part on the level of health improvements in the population they serve may be worth exploring. Several countries have successfully involved their medical facilities in preventive efforts [40-46]. Research using appropriate time-series cross-sectional methods and carefully constructed county-level covariates for all the potential key determinants is a priority for future work. It is only when this work is undertaken that a coherent assessment of the contributions of different distal and proximal factors will be available.

This study is largely descriptive, but because it uses a statistical model to estimate age-specific mortality at the county level, it has important limitations. In counties with small populations, life expectancy estimates are substantially informed by patterns in adjacent counties as well as levels of income, education, and racial composition. Our estimation procedure generates substantial uncertainty intervals. Underlying levels of mortality risk may in some small communities be substantially higher or lower than we estimate using our approach. Despite this important limitation, the use of mixed effects Poisson regression with spatial correlation of random effects combined with Gaussian Process Regression yields estimates with much narrower uncertainty intervals for small areas than uncertainty intervals generated using the binomial distribution and the normal approximation of binomial distribution of the observed data solely on the basis of sampling error. Alternative geospatial models have been proposed [16,47]. It will be important in future research to subject a range of alternative geospatial modeling strategies to rigorous out-of-sample predictive validity testing. It is also important to note that while life expectancy at birth is constructed to summarize period age-specific mortality rates, age-specific mortality rates in the youngest age groups and the changes therein have a much bigger impact on life expectancy at birth. To address this issue, we have examined the trends of standard deviation of the age-specific mortality rates in logarithmic scale. All age groups used in this study (18 out of the 18 five-year age groups) saw increases in the standard deviation of death rates in log scale. Therefore, increasing inequalities exist among all age groups.

Kindig and Cheng [16] recently reported that 42.8% of counties observed increases in female age-standardized death rates from the period of 1992–1996 to the period of 2002–2006. It is important to note that choice of standard population distribution could potentially alter the conclusion drawn from the comparison. We believe life expectancy, at birth or by age, is a more appropriate measurement in looking at the changes in mortality over time and across countries. When we use our life expectancy at birth generated in this study, we find many fewer counties have had statistically significant declines in life expectancy. Kindig and Cheng model directly the change in the age-standardized rate using fixed effects and state and county random effects. They report increases without indicating whether these changes are statistically significant or not. In applying the same methodology to the same time periods as Kindig and Cheng, we found that 30.4% of counties experienced an increase in age-standardized death rates for females, but the increase was significant in only 2.4%. In addition, our methods are not the same, as we first estimate age-specific death rates and then compute full life tables with uncertainty. We have noted that the number of counties with significant declines in female life expectancy has attenuated since 2002. It is also important to note that we have a longer time period compared to Kindig and Cheng [16], Kulkarni et al. [11], and Murray et al. [9]. Indeed, our longer time period and the use of more updated population estimates in our denominators enabled us to produce a more robust conclusion on the changes in mortality and inequality over time.

For nearly two decades, studies have drawn attention to wide disparities in life expectancy at the county level. Despite some attention and policy discussion, disparities continue to increase. Simply put, as a nation, the United States has failed to make any progress in reducing disparities at the county level, even though national life expectancy has increased. Large numbers of communities are being left behind; they are not seeing any increase in life expectancy. New strategies are needed to address this growing problem. While further understanding of the social, economic, and cultural distal determinants of health may provide critical insights, we also believe that a focus on modifiable behavioral, environmental, and metabolic risk factors provides a strategy that could work in the shorter term as well.

## Competing interests

All authors declare that they have no competing interests and therefore have nothing to declare with the exception of stating our core grant funding from the state of Washington.

## Authors’ contributions

CJLM, AES, and HW developed and applied the model to estimate mortality by age, sex, and race by county. AHM and CJLM designed the overall study and analytical strategy. AHM and CJLM wrote the first draft and revised the paper. All authors have read and approved the final manuscript.

## Supplementary Material

Additional file 1 Life expectancy for both sexes in every county across all years of the study (1985–2010). Click here for file
